# Federated learning for describing COVID-19 patients and hospital outcomes: An unCoVer analysis

**DOI:** 10.1093/eurpub/ckac131.254

**Published:** 2022-10-25

**Authors:** JL Peñalvo, E Mertens, J Cottam, V Berrozpe-Maldonado, D Fernández-Lobón, O Solarte-Pabón, E Menasalvas

**Affiliations:** 1 Unit of Non-Communicable Diseases, Institute of Tropical Medicine, Antwerp, Belgium; 2 Biomedical Engineering and Telemedicine, Universidad Politécnica de Madrid, Madrid, Spain

## Abstract

**Background:**

Since the onset of the pandemic, the unCoVer network has been identifying real-world data from EMR of hospitalised patients with COVID-19 across countries. These heterogeneous data are integrated into a multi-user data repository operated through Opal/DataSHIELD, an interoperable open-source server application, providing privacy-preserving access to individual-level information for federated data analyses.

**Methods:**

unCoVer’s federated data platform provided access to EMR collected between 02/2020 - 04/2022 from 6 hospitals in Bosnia and Herzegovina (1), Romania (2), Spain (2), and Turkey (1) for a total of 14,236 patients. Demographics, and co-morbidities at admission, length of hospital stay and intensive care (ICU) needs, are presented according to the patients’ status at discharge.

**Results:**

A total of 11,248 (79.0%) of all patients reviewed recovered from COVID-19 after an average 11.5 (SD 10.8) days hospitalised, with only 4.09% of patients needing ICU. A smaller proportion of patients were transferred (5.93%), and 2143 (15.1%) were considered in-hospital deaths after an average 11.6 (SD 10.5) days in the hospital where most (81.2%) needed ICU. Recovered patients had a mean age of 57.7 (SD 16.3) years old, and gender neutral (51.2% men), in contrast to deceased patients that were 74.2 (SD 12.4) years old (59.7% men). Current smoking was infrequent for both recovered or deceased patients (3.27%, and 2.83%, respectively). Cardiometabolic conditions were less commonly reported among later recovered patients in comparison with deceased patients: obesity (10.7% vs 12.1%), diabetes (15.9% vs 27.4%), hypertension (23.2% vs 42.7%), and CVD (9.33% vs 44.9%). Chronic pulmonary disease was also more frequent among deceased patients (10.3% vs 18.1%).

**Conclusions:**

Characteristics of hospitalised COVID-19 patients differ according to outcomes at discharge with more in-hospital death reported among older, chronic patients across 6 hospitals in 4 countries.

**Key messages:**

• Federated analyses provide unique opportunities for robust results by privacy-preserving accessing individual-level data from heterogeneous data sources.

• The unCoVer network aims to demonstrate the usability of the infrastructure to address research questions related to the COVID-19 while extending the concept to other clinical areas.

